# Advances in Organic–Inorganic Hybrid Latex Particles via In Situ Emulsion Polymerization

**DOI:** 10.3390/polym15142995

**Published:** 2023-07-10

**Authors:** Yubin Wang, Baojiang Sun, Zhiwei Hao, Jianhua Zhang

**Affiliations:** 1 School of Petroleum Engineering, China University of Petroleum (East China), Qingdao 266580, China; 2 CNPC Engineering Technology Research Co., Ltd., Tianjin 300451, China; 3 Department of Polymer Science and Engineering, Key Laboratory of Systems Bioengineering of the Ministry of Education, School of Chemical Engineering and Technology, Tianjin University, Tianjin 300350, China; 4 Tianjin Key Laboratory of Membrane Science and Desalination Technology, Tianjin University, Tianjin 300072, China

**Keywords:** polymer nanocomposites, hybrid latex particles, in situ emulsion polymerization, Pickering emulsion

## Abstract

Hybrid latex particles combine the unique properties of inorganic nano/micro particles with the inherent properties of polymers, exhibiting tremendous potential for a variety of applications. Recent years have witnessed an increased interest in the design and preparation of hybrid latex particles with well-defined size, structure and morphology. Due to its simplicity, versatility and environmental friendliness, the in situ (Pickering) emulsion polymerization has been demonstrated to be a powerful approach for the large-scale preparation of hybrid latex particles. In this review, the strategies and applications of in situ (Pickering) emulsion polymerization for the preparation of hybrid latex particles are systematically summarized. A particular focus is placed on the strategies for the preparation of hybrid latex particles with enhanced properties and well-defined core–shell, yolk–shell, multinuclear, raspberry-like, dumbbell-shaped, multipod-like or armored morphologies. We hope that the considerable advances, examples and principles presented in this review can motivate future contributions to provide a deeper understanding of current preparation technologies, develop new processes, and enable further exploitation of hybrid latex particles with outstanding characteristics and properties.

## 1. Introduction

During the last several decades, we have witnessed a very rapid development and evolution of composite materials, which are a type of material consisting of two or more constituent components with different physicochemical characteristics and distinct boundaries. The composite materials combine the advantages of constituent materials and exhibit markedly different characteristics from the individual components [1,2,3,4]. Composite materials have long been employed in the history of the development of human society. For example, mud and straw were combined to form bricks with enhanced and reinforced strength several thousand years ago. Concrete has been widely used for buildings, which is actually a composite of stones in a matrix of cement. However, the composite materials came to the attention of industries only in the 1960s, with the innovation and creation of polymer-based composites [4,5,6,7,8,9].

Polymer composite materials are a kind of composite material that physically or covalently incorporates nanosized particles or nanostructures into a matrix of cross-linked polymer networks [4,5,6,7,8,9], which have been developed as a popular means for the creation of novel materials with diverse functionality. The flexible macromolecular chains and the rigid nanofillers can be considered as mortar and bricks. The flexible macromolecular chains as mortars can connect, host and integrate the bricks to maintain the structural integrity. Meanwhile the introduced rigid nanofillers as bricks render new functionalities to the final polymer composites. The incorporation of nanomaterials into polymer matrixes has been demonstrated to be able to render new functionalities to the composites [10,11,12,13,14]. For example, the introduction of nanofillers into polymers can improve mechanical properties of polymers. Usuki et al. significantly improved the tensile strength of the nylon-6 network by introduction of montmorillonite, a type of nanoscale natural silicate mineral [15]. Moreover, the incorporation of functional nanomaterials into polymeric networks can impart superior physicochemical properties absent in the individual components. For example, the entrapment of magnetic nanofillers into polymer networks can impart the resulting polymer nanocomposites with magnetic character [16]. Similarly, incorporation of gold or silver nanofillers into a polymer matrix will render excellent antimicrobial properties [17,18]. The introduction of gold nanorods into polymer matrixes can be used to manipulate optical properties [19]. The fabrication of polymer composite materials offers an efficient route to enhance their physicochemical properties and thus expand their application scopes.

Hybrid latex particles are one of the typical and widely used polymer composite materials, which have been widely used in paints, adhesives, binders, carpet backing, paper coatings, textile treatments, pharmaceutics and so on [2,3,20,21,22,23]. The hybrid latex particles are a sort of latex particle containing a high molar mass polymer phase and inorganic material phase, and which are usually a stable colloidal dispersion of polymer particles dispersed or suspended in water. The particles often possess a spherical shape ranging in size from dozens of nanometers to a few microns in diameter. The scope of these hybrid latex particles containing organic and inorganic materials in applications is much broader than that of polymer latex particles.

**Scheme 1:**
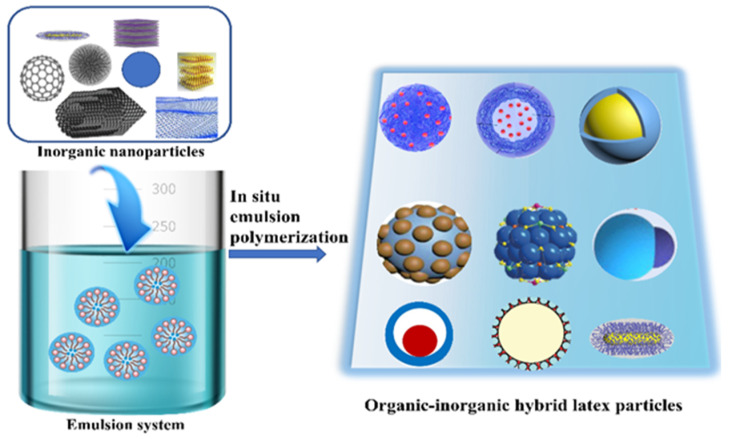
Schematic illustration of in situ emulsion polymerization to prepare organic–inorganic hybrid latex particles.

The methods and strategies involving either chemical or physicochemical approaches for the preparation of hybrid latex particles have been designed and reported during the last several decades [2,3,22,23,24]. The strategies mainly include heterocoagulation, layer-by-layer assembly techniques, and in situ emulsion polymerization. One of the widely used methods to prepare hybrid latex particles is the physical heterocoagulation process. In this process, two or more types of particles with different composition and/or size are prepared previously in a separate step and are then coagulated in a controlled way via electrostatic forces, hydrophobic interactions, hydrogen bond interactions and/or specific molecular recognition. For example, different particles with opposite charge can undergo physical adsorption by electrostatic interactions, which are widely used in the heterocoagulation process. Usually, the positively charged inorganic particles are pre-prepared and then slowly added into a surplus of negatively charged latex particles. The charge reversal of the inorganic particles takes place, inducing the adhesion of inorganic particles onto the surface of pre-existing polymer latex particles [23,24]. As an easy to carry out and very versatile method to prepare hybrid latex particles, the layer-by-layer assembly technique can be considered as a repetitive extension of heterocoagulation. The driving forces of the assembly process mainly include electrostatic interactions, hydrophobic interactions and hydrogen bonding interactions. Especially layer-by-layer assembly driven by electrostatic interactions has been widely used and studied in the fabrication of micro- and nanometer-sized latex particles [23,24,25]. By alternating layers of positively and negatively charged particles and/or polyelectrolytes, the different components were sequentially adsorbed onto the surface of the pre-formed particles. Compared with the abovementioned approaches, the in situ emulsion polymerization represents the simplest type of approach to the large-scale preparation of hybrid latex particles with specific morphologies. In a typical in situ emulsion polymerization process to hybrid latex particles, the inorganic nanoparticles are dispersed in a precursor solution of monomer, dispersing medium, emulsifier, and water-soluble initiator and then the hybrid latex micro/nanoparticles are obtained after the polymerization of the monomer via standard emulsion polymerization techniques [2,3,22,24,26]. Recently, the in situ emulsion polymerization, especially the in situ Pickering emulsion polymerization, has been demonstrated to be a powerful approach for the preparation of hybrid latex particles with enhanced properties and well-defined core–shell, yolk–shell, multinuclear, raspberry-like, dumbbell-shaped, multipod-like or armored morphologies [2,22,27,28,29,30,31,32], as shown in Scheme 1. However, as far as we know, little work has been performed to discuss the progress and development of hybrid latex particles prepared via in situ emulsion polymerization. This review will summarize the advances in the field of hybrid latex particles, which will place particular focus on the preparation strategies of in situ (Pickering) emulsion polymerization.

## 2. Hybrid Latex Particles by In Situ Emulsion Polymerization

The past several decades have witnessed a surge of efforts to incorporate various kinds of nanomaterials into polymer matrixes to create novel polymer composite materials with superior properties and desirable functionalities for various applications [14,33,34]. The hybrid latex particles are typical examples of polymer composite materials in which inorganic nanoparticles (NPs) were integrated into, or dispersed within, polymer latex particles. The integration of various inorganic NPs into polymer latex particles has become a powerful tool for the development of high-performance polymeric latexes. A range of inorganic NPs, such as silica-based NPs (silica, silicates, mica), carbon-based nanomaterials (carbon spheres, carbon nanotubes, graphene, graphene oxide, fullerene) and metal/metal-oxide nanoparticles (gold, silver, alumina, copper, platinum, iron oxides and titanium oxides) have been integrated within the polymer latex particles to obtain hybrid latex particles with superior physicochemical properties and tailored functionalities and high performances for various applications [3,11,12,13,14,29,30,31,34]. Based on their morphology and structure, the inorganic NPs can be categorized into nanospheres, nanofibers and nanoplatelets. Various nanoparticles of spherical shape are the most common nanomaterials, which can improve the performance of polymers and provide the resulting polymer nanocomposites with novel properties. Nanofibrous fillers, such as carbon nanotubes, metal nanowires and cellulose nanocrystals, etc., are another common type of nanomaterial. This type of NP can usually be treated as a nanowire of certain flexibility that can be bent to a limited degree. Nanoplatelets or nanosheets are other types of nanomaterials that usually have thicknesses of several to tens of nanometers and diameters of tens to thousands of nanometers, which have exhibited extraordinary effects on the mechanical performance of the final polymers.

Hybrid latex particles can be made by the combination of various inorganic NPs and polymer latex particles via various mechanisms [2,10,11,12,13,14,22,23,24,25,33,34]. One of the most simple and general methods to prepare hybrid latex particles is in situ emulsion polymerization, in which a polymer-forming monomer solution is polymerized in a dispersion of pre-formed NPs. As a typical heterogeneous polymerization, unlike homogeneous bulk polymerization or solution polymerization, the emulsion polymerization can provide some advantages, such as mild reaction conditions, low viscosity and thus improved heat and mass transfer, high conversion of monomers, minimization of separation and recycling as well as enhanced environmental benefits due to the use of water as a medium [35,36]. In situ emulsion polymerization has been proven to serve as an effective way to prepare multifunctional hybrid latex particles with various architectures and properties [37]. The efficient dispersion of the inorganic NPs in the solution is very important, because the sedimentation processes may proceed very obviously, leading to the failure of polymerization or poorly-dispersed NPs in the subsequently formed polymer. However, it is often very difficult for inorganic NPs with high surface energy to be redispersed within the polymer emulsion at the nanoscale through simple mixing. In conventional emulsion polymerization, surfactants are used to decrease interfacial tension between hydrophobic monomer and water, stabilize the latex and generate micelles. However, when it comes to in situ emulsion polymerization, surfactants or other ligands were often used to enhance the particle dispersion by binding to the nanofillers’ surfaces. In addition, the inorganic NP surfaces were often modified to improve particle dispersion and offer high compatibility with the emulsion solutions. It is especially worth pointing out that the during the in situ emulsion polymerization process, the surfactant molecules do not participate in the polymerization and sometimes the inorganic NPs are not directly linked to the subsequent polymer. In sum, these strategies have opened up new possibilities in developing in situ emulsion polymerization for the preparation of various hybrid latex particles, which will be described below.

### 2.1. Silica-Doped Hybrid Latex Particles

**Figure 1:**
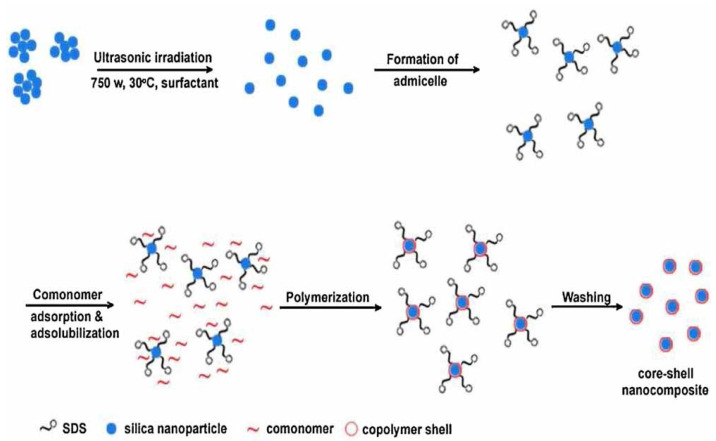
Schematic illustration of the proposed formation mechanism of copolymer encapsulated SiO_2_ through ultrasonically assisted in situ emulsion polymerization, reproduced with permission from Wiley [42].

Various kinds of silicon NPs possess some unique advantages, such as low cost, chemical inertia, high thermal and mechanical stability, facile surface modification and easy functionalization, and have been extensively studied as fillers in polymer composite materials [6,38,39,40,41]. During the past decade, great progress has been made in the development and application of in situ emulsion polymerization to prepare silica-doped hybrid latex particles. For example, solid silica and mesoporous silica NPs have also been widely used to prepare various hybrid latex particles via in situ emulsion polymerization. For example, Shim et al. [42], applied an ultrasonically assisted in situ emulsion polymerization to prepare electrically conducting copolymer poly(aniline-*co*-*p*-phenylenediamine) (poly(Ani-*co*-*p*PD)) and silica (SiO_2_) nanocomposites with sodium dodecyl sulfate (SDS) as an emulsifier. They demonstrated that the ultrasonic irradiation could reduce the aggregation of SiO_2_ NPs and the ultrasonically assisted in situ emulsion polymerization can solve problems in the dispersion and stabilization of SiO_2_ NPs in the emulsion system. The polymerization then occurred at the interfaces between nanometer-sized oil droplets and the water phase, as shown in Figure 1. Due to the interaction between copolymer chains and SiO_2_, the generated copolymer particles would diffuse continuously to the surface of the SiO_2_ to coat the SiO_2_ nanoparticles, leading to the formation of the poly(Ani-*co*-*p*PD)/SiO_2_ nanocomposite with a core–shell structure. Wang et al. [43] prepared a kind of multifunctional SiO_2_/P(St-KH570) nanocomposite with core–shell structure through in situ emulsion polymerization of styrene (St) and 3-methactyloxylpropyltrimethoxyl silane (KH570) in the sodium metasilicate solution. They also reported a type of monodisperse PSt/SiO_2_ composite nanoparticle with a core–shell structure by in situ emulsion polymerization of St on the surface of oleic acid grafted silica nanoparticles [44]. Jalali-Arani et al. [45] used a silane coupling agent (3-(trimethoxysilyl) propyl methacrylate) bearing unsaturated carbon double bonds to modified silica nanoparticles. The hydrophobicity and the ability to take part in free radical polymerization of the modified nanosilica can be obviously increased. The hybrid poly (methyl methacrylate-*co*-butylacrylate)/nanosilica nanocomposites with core–shell structures were synthesized by a soap-free seeded emulsion polymerization in the presence of ionic comonomers (sodium salt of styrene sulfonic acid or/and potassium methacrylate). The ionic comonomers can stabilize the NPs and create ionic charge groups on their surface. The sodium salt of styrene sulfonic acid can decrease the particle size more effectively compared with the potassium salt of methacrylic acid, which may be due to that the sulfonic-based salts are more effective in decreasing the particle size and the particle size distribution than the carboxylic-based salts. More interestingly, they found that the concurrent presence of carboxylic and sulfonic salts can not only successfully stabilize the hybrid nanoparticles, but also generate cationic–anionic ionic groups on the surface of hybrid particles with much smaller size, due to the trade-off between effects of electrostatic repulsive forces and the medium’s ionic strength on the average particle size. Tayebi et al. [46] synthesized polyacrylate-encapsulated nanosilica via in situ emulsion polymerization using nonyl phenol polyethylene glycol ether (NP7). The surface of SiO_2_ particles were modified with thermoresponsive nonionic surfactant NP7 and then the emulsion polymerization of the acrylic monomers was initiated on the surface of the NP7-modified SiO_2_ in the presence of sodium dodecyl benzenesulfonate. The mechanical properties and thermal stability of the final nanocomposites were remarkably improved owing to the uniform dispersion of the non-agglomerated SiO_2_. Few studies have reported nanocomposites consisting of polymer and mesoporous silica. Recently, Zhang et al. [47] introduced the mesoporous molecular sieve SBA-15 into a polystyrene matrix via in situ emulsion polymerization of styrene. A small amount of the mesoporous silica with an ordered structure, large surface area and uniform pore size can be well dispersed within the polystyrene matrix, leading to an improvement in glass transition temperature, thermal stability and mechanical performance of the polymer. The results also indicated that the mesoporous silica could serve as a good reinforcing agent for polystyrene.

**Figure 2:**
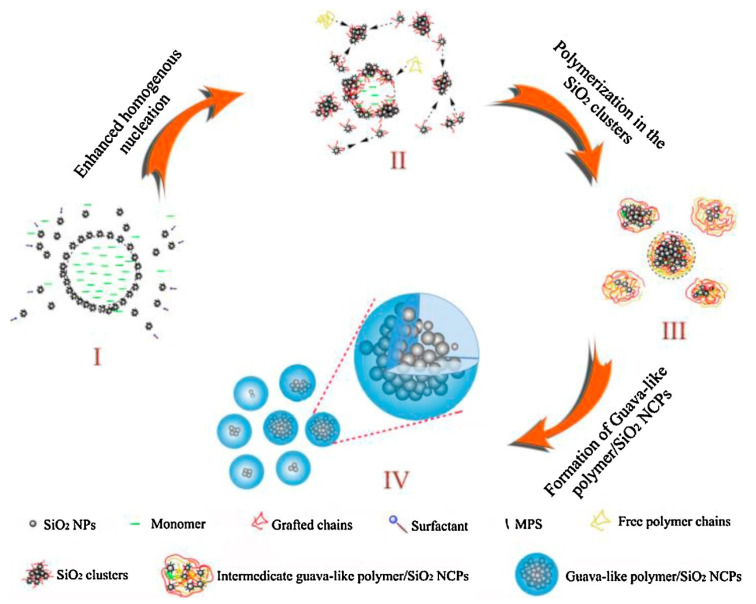
Schematic representation of the formation mechanism of guava-like polymer/SiO_2_ nanocomposites through in situ emulsion polymerization in the presence of 3-(trimethoxysilyl) propyl methacrylate (MPS)-modified SiO_2_ NPs, reproduced with permission from Elsevier [50].

**Figure 3:**
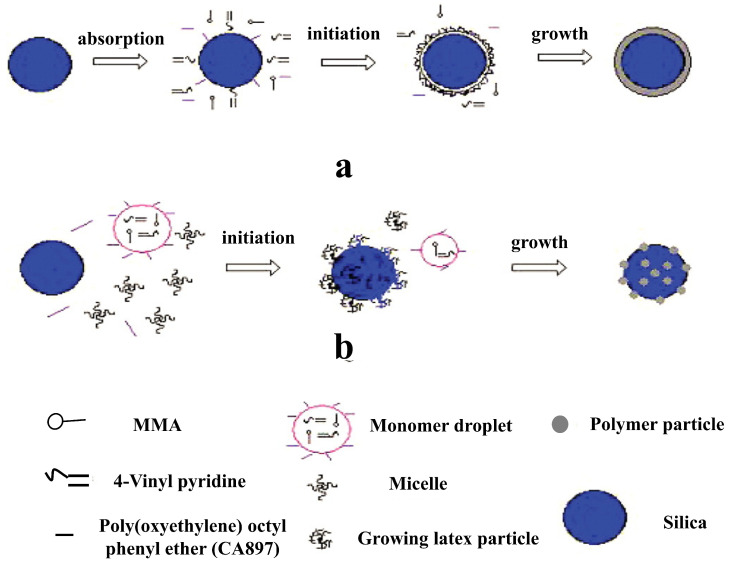
Schematic illustrations of formation mechanisms for composite particles with (**a**) core–shell morphology and (**b**) raspberry-like morphology, reproduced with permission from Wiley [57].

Ravaine et al. [48] synthesized a kind of snowman-like or raspberry-like polystyrene/SiO_2_ nanocomposite via in situ emulsion polymerization of styrene in the presence of SiO_2_ surface-modified by adsorption of an oxyethylene-based macromonomer. The morphology of the final hybrid polystyrene/SiO_2_ nanocomposites can be adjusted by varying different experimental parameters, especially the ratio between the number of the surface-modified SiO_2_ seeds and the number of the polystyrene nodules. These researchers also synthesized daisy-shaped and multipod-like silica/polystyrene nanocomposites using in situ emulsion polymerization according to a similar procedure as mentioned above [49]. As shown in Figure 2, in situ emulsion polymerization was used to prepared guava-like polymer/SiO_2_ nanocomposites and their formation mechanism was revealed [50]. Additionally, 3-(trimethoxysilyl) propyl methacrylate (MPS) was first used to modify SiO_2_ nanoparticles (NPs) and the resulting MPS-modified SiO_2_ nanoparticles were pre-dispersed in the aqueous continuous phase of the monomer emulsion (Styrene (St) or/and methyl methacrylate (MMA)). They found that some of the SiO_2_ NPs as colloidal stabilizers can be adsorbed onto the outer surface of the monomer droplets, similar to Pickering emulsion systems. After initiation, the generated oligomeric radicals were mostly captured by the micelles and underwent micelle nucleation. The oligomeric radicals of MMA with high radical activity in in situ emulsion polymerization of St can react with vinyl groups on the outer surface of MPS-modified SiO_2_ NPs, increasing the hydrophobicity and then leading to the aggregation of SiO_2_ NPs to form a cluster. The addition of MPS-modified SiO_2_ NPs could promote the occurrence of homogenous nucleation, leading to the formation of more guava-like polymer/SiO_2_ nanocomposites. Yang et al. [51] also prepared a kind of monodispersed silica-polymer core–shell nanosphere by the use of MPS-modified SiO_2_ NPs via in situ emulsion polymerization of MMA. They found that the thickness of polymer shells depended on the amount of monomer and MPS-modified SiO_2_ NPs as well as the concentration of emulsifiers. Bourgeat-Lami et al. [52] also reported hybrid dissymmetrical snowman- and dumbbell-like silica/polymer colloidal particles through in situ emulsion polymerization of methyl methacrylate or styrene. The bicationic initiator 2,2′-azobis(N,N′-dimethyleneisobutyramidine) dihydrochloride was anchored on the silica surface, due to electrostatic interaction between the silica surface and the cationic amidinium groups of the diazoic compound. Anisotropic particles with a poly(methyl methacrylate) or polystyrene latex particle attached to a single silica sphere were successfully obtained in in situ emulsion polymerization of methyl methacrylate or styrene by the use of a mixture of nonionic and anionic surfactants in the presence of initiator-anchored particles. They also synthesized a type of silica-poly(methyl methacrylate) latex particle with raspberry-like or core–shell morphology through in situ emulsion polymerization of methyl methacrylate in the presence of silica beads as the seed [53]. They also investigated the role of initiators in the synthesis of silica/poly(methyl methacrylate) nanocomposite latex particles through in situ emulsion polymerization [54]. Three different initiators including 2,2′-azobis(2-amidinopropane) dihydrochloride (AIBA) as cationic initiator, potassium persulfate (KPS) as anionic initiator and azobis(isobutyronitrile) (AIBN) as nonionic initiator were used to evaluate the role of the surface charge of the hydrophilic silica on the coating reaction. AIBA was found to be adsorbed on the silica surface owing to electrostatic interactions, while the anionic and the nonionic initiators did not adsorb to silica under the same conditions. The results indicated that the polymer content and the coating efficiency greatly depend on the nature of the initiator and the cationic initiator can significantly decrease the amount of free polymer. Similarly, Han et al. [55] used 1,3,5,7-tetravinyl-1,3,5,7-tetramethylcyclotetrasiloxane (Vi-D_4_) with multi-silanol groups and active multi-vinyl groups as modifier to treat the inorganic SiO_2_ surface. Additionally, the in situ emulsion polymerization of styrene was used to prepare polymer/SiO_2_ nanocomposites in the presence of Vi-D_4_-modified silica particles as seeds and sodium dodecyl benzene sulfonate as emulsifier. The resulting nanocomposites have obvious core–shell structure, a single core in each composite particle. Dan et al. [56] synthesized a kind of poly(methyl methacrylate) (PMMA)/SiO_2_ composite particle through in situ emulsion polymerization. The dispersion of PMMA/SiO_2_ composite in latex was demonstrated to be better than that of silica particles, as the grafting of PMMA chains onto the surface of silica particles can alleviate the aggregation of silica particles and decrease the size of the dispersion phase. Wu et al. [57] applied the acid–base interaction between the silanol groups of the silica surface and the amino group of 4-vinyl pyridine to synthesize SiO_2_/PMMA composite particles via emulsion copolymerization. As shown in Figure 3, the composite particles could be multicore–shell, raspberry-like or normal core–shell morphology on the basis of the emulsifier content, monomer/silica ratio, silica particle size, monomer feed method, and so forth. They verified that in situ emulsion polymerization is very simple and helpful in the fabrication of various polymer nanocomposites with controllable morphology. They also synthesized a series of SiO_2_/poly(styrene-*co*-butyl acrylate) nanocomposite microspheres with various morphologies by in situ mini-emulsion polymerization [58]. The results showed that the incorporation of the soft monomer (butyl acrylate) can facilitate the formation of the multicore–shell structure.

Buhin et al. [59] synthesized polyacrylate/silica (pyrogenic and colloidal silica fillers with various particle morphologies) nanocomposites via in situ emulsion polymerization with nonionic emulsifier in order to obtain good dispersibility of nanosilica in the polyacrylate matrix. The effect of morphology and concentration of silica nanofiller on emulsion particle size as well as on thermal and mechanical properties of the nanocomposite films were investigated in detail. They demonstrated that pyrogenic and colloidal silica fillers with significantly different morphology can cause a big difference in the emulsion particle size, thermal stability and mechanical properties. Similarly, polyvinyl acetate (PVAc)/colloidal silica nanocomposites for wood adhesives were synthesized via in situ emulsion polymerization of vinyl acetate monomer in the presence of polyvinyl alcohol. The resulting PVAc/colloidal silica nanocomposites exhibited good thermal stability and thermal barrier properties as well as an obviously enhanced adhesion strength for wood specimen bonding [60]. Chen et al. [61] also prepared PVAc/colloidal silica nanocomposites by use of vinyl-functionalized SiO_2_ NPs through in situ emulsion polymerization. Zhao et al. [62] prepared organic nano-silica/polyacrylate composite by combining a sol-gel method and acrylate-based in situ emulsion polymerization. Asadinezhad et al. [63] synthesized a kind of nanocomposite consisting of poly (methyl methacrylate-*co*-butyl acrylate-*co*-acrylic acid)/nanosilica by seeded semi-batch in situ emulsion polymerization. They found that, after introducing nanosilica NPs, the reaction conditions (pH and temperature) woulddetermine whether the hybrid latexes could achieve minimal coagulum level and maintain long-lasting stability. Romo-Uribe et al. [64,65] also synthesized a series of polyacrylate/SiO_2_ nanocomposites by in situ emulsion polymerization in the aqueous phase using as-received SiO_2_ NPs. The influence of the concentration of SiO_2_ nanoparticles on yield, coagulum, kinetics, and thermomechanical properties was investigated. The results indicated that the presence of SiO_2_ NPs can slow down the polymerization and decrease the final conversion. However, the introduction of SiO_2_ NPs can also induce higher thermal stability and increase the Young’s modulus. The conducting polypyrrole/SiO_2_ nanocomposites were prepared by an in situ emulsion polymerization method. To achieve high compatibility between polypyrrole and SiO_2_, 3-aminopropyltriethoxysilane was used to modify SiO_2_ spherical NPs [66]. The resulting polypyrrole/SiO_2_ hybrid latex and the resulting films exhibited high electrical conductivity.

### 2.2. Carbon-Doped Hybrid Latex Particles

As one of the most abundant elements, carbon can be found in all living things. Due to its unique physicochemical properties, including high mechanical strength, high corrosion resistance, high specific surface area and high electrical conductivity, carbon nanomaterials as the most extensively studied materials, have gained worldwide popularity [67,68,69]. As a result, extensive research efforts have introduced various carbon nanomaterials with favorable properties into polymer matrixes to create nanocomposite materials with enhanced physical and mechanical properties for a variety of applications [34,70,71,72,73,74,75,76,77,78,79,80]. Among various carbon-based nanomaterials, carbon nanotube and graphene-based NPs have been extensively explored to develop functional polymers with markedly enhanced mechanical, electrical and thermal properties [72,73,74,75]. However, the key problem of the practical use of carbon-based NPs is their poor dispersibility in water, organic solvents and the polymer matrix [72,73,74,75,81,82]. Generally, some pretreatments or surface modification with surfactants, polymers or proteins are often used to prompt the dispersion of carbon-based NPs. Recently, hybrid latex particles consisting of various polymer and carbon-based NPs have been prepared by use of in situ emulsion polymerization. Typically, the hydroxylated and carboxylated multi-walled carbon nanotubes (CNTs) were dispersed into water in the presence of the reactive emulsifier allyloxy decyl polyoxyethylene ether ammonium sulfate (KL-10). Then, the monomer solution and initiator solution were added. After emulsification, the in situ emulsion polymerization was carried out to obtain styrene–acrylic emulsion/carbon nanotube nanocomposites (S-A/CNTs) [83]. The as-synthesized composite latex could be employed as a high-performance conductive coating or an electrostatic shielding coating [83]. Qiu et al. [84] demonstrated that the ultrasonic irradiation and ultrasonic cavitation can not only generate radicals to initiate the polymerization without any added initiator, but also exert an outstanding dispersion effect on CNTs in aqueous solution, leading to breakage of the aggregation and entanglement of CNTs with ultrasound cavitation. Thus, the ultrasonically initiated in situ emulsion polymerization of monomer n-butyl acrylate and methyl methacrylate in the presence of CNTs provides an effective surface modification method for CNTs with high surface energy. Kim et al. [85] prepared polyaniline/activated carbon composites via in situ emulsion polymerization by use of dodecyl benzenesulfonic acid (DBSA). The effect of DBSA on the polyaniline/activated carbon composites was investigated with various molar concentrations of DBSA, and they found DBSA can play a role as both surfactant and dopant in the process of PANI synthesis, leading to various electrochemical behaviors of the composites. Gomes et al. [86] utilized in situ emulsion polymerization to synthesize polystyrene/CNTs polymer brush composites. To enable superior dispersion and high attachment to the polymer matrix during the polymerization, the CNTs were surface modified using oleic acid and subsequent organosilane chemistry. The morphological characterization confirmed excellent integration of the CNTs within the polystyrene matrix and the resulting polystyrene/CNT composites displayed great improvement in thermal stability with increase in CNT concentration.

**Figure 4:**
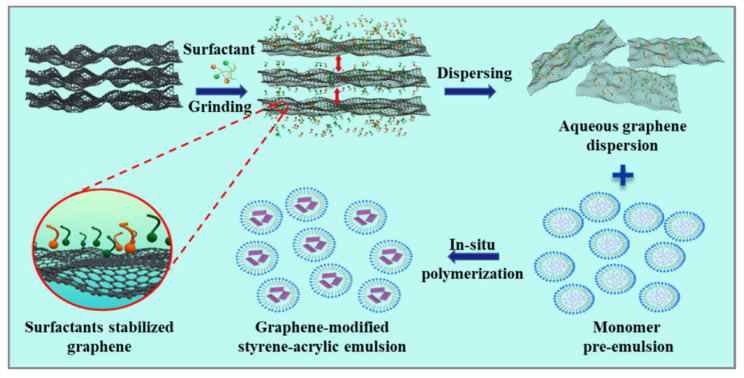
Schematic illustration of in situ emulsion polymerization to prepare graphene-modified styrene–acrylic emulsion particles, reproduced with permission from MDPI [89].

In comparison with one-dimensional CNTs, the two-dimensional graphene-based nanomaterials, including graphene, graphene oxide (GO) and reduced graphene oxide (rGO), have exhibited some distinctive properties, such as large surface area, high aspect ratio, outstanding mechanical properties, excellent thermal and electrical conductivity as well as good optical transparency [67,69,87,88]. Thus, they have also been extensively explored to prepare functional nanocomposites. For example, Qian et al. [89] introduced the graphene nanopowder into waterborne styrofoam coating by in situ emulsion polymerization, which was expected to resolve the problem of poor interface compatibility between graphene and the polymer resin. As shown in Figure 4, the surfactants were demonstrated to be able to enhance the dispersion of graphene in water and then the obtained graphene aqueous dispersion was mixed with the pre-polymerization of styrene–acrylic emulsion monomer, leading to the formation of graphene-modified styrene–acrylic emulsion. After in situ emulsion polymerization, the graphene was successfully loaded into the styrene–acrylic emulsion. The physical and chemical properties of the resulting graphene-modified emulsion were investigated, and the results showed that the styrene–acrylic emulsion with 4 wt% aqueous graphene dispersions displayed a significantly improved dispersion stability, enhanced water and oxygen resistance, as well as good electromagnetic shielding performance. Similarly, Xu et al. [90] reported a facile and rapid preparation of reduced graphene oxide nanosheets–polystyrene nanocomposites via in situ emulsion polymerization for a variety of applications. Firstly, the aqueous graphene dispersions were mixed with styrene monomer and surfactant SDS under ultrasonication. After the formation of styrene-linked graphene micelles, the polymerization of styrene in the adsorbed micelles was initiated to produce the graphene oxide nanosheets–polystyrene latex particles with a high conductivity level. Kim et al. [91,92] reported a similar in situ emulsion polymerization to prepare polyaniline (PANI) and PPy nanocomposites containing 5 wt% of GO with outstanding electrical properties. In this study, they simultaneously applied dodecyl benzene sulfonic acid (DBSA) as an emulsifier and protonating agent to initiate the formation of PANI and PPy during the polymerization reaction. Their studies indicated that the in situ emulsion polymerization can provide mild experimental conditions to achieve better structural, thermal, and electroconductive properties. The systematic evaluation and comparison of the thermal, electrical, and electroconductive properties of the resulting nanocomposites, indicated that the direct introduction of GO and other nanofillers into conductive polymers via the in situ emulsion polymerization process by the use of DBSA as a surfactant, achieved nanocomposites with promising properties for various semiconductive applications. The groups of Hussain [93] and Yao [94] also used DBSA as an emulsifier and protonating agent to prepare polypyrrole/graphene nanocomposites with harnessed conductivities via a modified in situ emulsion polymerization under mild conditions. Janin et al. [95] reported a kind of in situ emulsion cationic polymerization of isoprene to prepare polyisoprene/GO nanocomposites. The allyltrimethoxysilane (ATMS) was firstly grafted onto the surface of GO sheets and then the ATMS-modified GO sheets were stably suspended in toluene. Subsequently, a highly water-dispersible Lewis acid surfactant-combined catalyst was prepared and used to initiate the emulsion polymerization of isoprene in a simple one-pot reaction in the presence of ATMS-modified GO sheets. Similarly, Xu et al. [96] prepared poly(vinyl acetate)-grafted graphene oxide (PVAc-g-GO)/PVAc nanocomposites by in situ emulsion polymerization of vinyl acetate with water as solvent at low temperature (10 °C). They found that the GO could be homogeneously dispersed in a water-based emulsion system and the inevitable agglomeration of GO during polymerization was effectively limited under low temperature conditions. PVAc-g-GO)/PVAc nanocomposites can be changed simply into PVA-g-GO/PVA nanocomposites through alcoholysis and offer an effective method to develop GO-based PVA nanocomposites with excellent properties for a wide range of applications. Tomovska et al. [97,98] first prepared highly concentrated and very stable reduced graphene oxide (rGO) aqueous dispersion by the use of polyvinyl pyrrolidone (PVP) as a polymeric surfactant to ensure colloidal stability to prevent aggregation. Subsequently, the stable rGO dispersions were used as a water phase in situ emulsion polymerization of methyl methacrylate (MMA) and butyl acrylate (BA) in a 50/50 wt% ratio. The resulting hybrid dispersions with decreased aggregation of rGO have shown excellent stability during storage of more than 6 months. The rGOs/polymer latexes can be used to prepare nanocomposite film after water evaporation. These tests indicated that the rGO can be uniformly dispersed in the polymer matrix.

**Figure 5:**
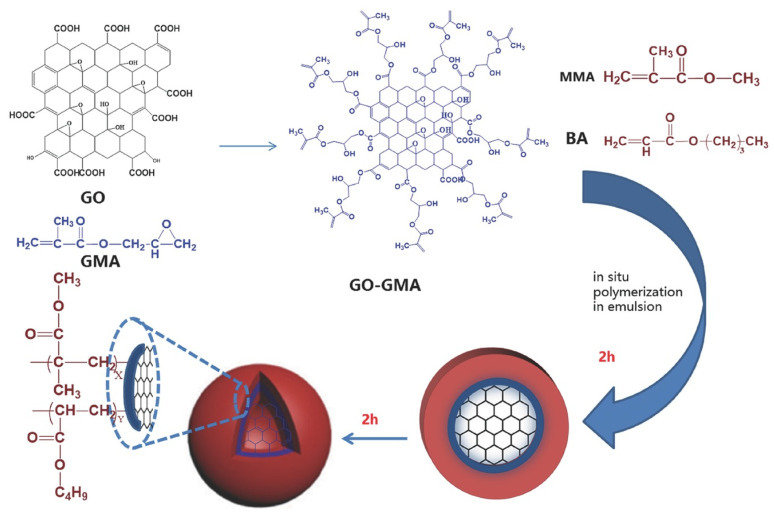
Schematic illustration of in situ emulsion polymerization to prepare core–shell GO-doped hybrid latex particles, reproduced with permission from Wiley [99].

Recently, a kind of core–shell nanocomposite with a graphene oxide (GO) core and a poly(methyl methacrylate/butyl acrylate) (MMA/BA) shell has been synthesized through in situ emulsion polymerization [99]. As shown in Figure 5, the vinyl groups were firstly introduced onto the GO through a chemical reaction between the epoxy group of glycidyl methacrylate (GMA) and the carboxyl groups from the edge of GO. The GO with vinyl groups can be used to copolymerize with MMA and BA during in situ emulsion polymerization. The resulting latex is homogeneous without any aggregation and keeps stable over 100 days at normal temperature, and can be applied as an ideal conductive adhesive possessing enhanced electrical conductivity in a high humidity atmosphere.

### 2.3. Metal-Doped Hybrid Latex Particles

The lack of thermal, magnetic, optical and electrical conductivity often severely limits the wide applications of polymers [100,101]. It is well known that metal and metal oxide materials often have magnetic, electrical and optical properties, which are not commonly found in polymeric materials. The integration of metal and metal oxide materials into polymer matrixes has demonstrated considerable potential to produce polymer composites with unique characteristics and tunable properties [6,100,101,102,103]. In the past several decades, all kinds of metal-based nanofillers and metal oxides, as well as metal alloys and salts with desirable physicochemical properties, have been introduced into emulsion polymerization to prepare hybrid latex particles [104,105,106,107,108].

**Figure 6:**
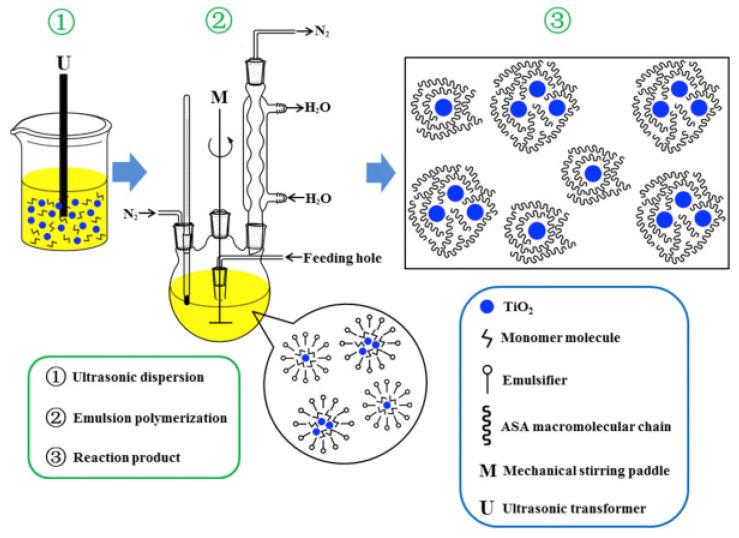
Schematic diagram for the preparation of TiO_2_/ASA hybrid latex particles via in situ emulsion polymerization, reproduced with permission from Elsevier [111].

Typically, titanium dioxide (TiO_2_) NPs as solid transition metal oxides have also been widely used to fabricate hybrid latex particles, due to their high chemical stability, excellent electronic conductivity, high thermal resistance, good corrosion and oxidation resistance, [109,110]. The introduction of TiO_2_ nanofillers into polymer matrixes has been demonstrated to be able to not only increase the stiffness, toughness and durability, but also impart conductivity, fire retardation and photocatalytic activity. However, the application of TiO_2_ nanofillers is restricted to a great degree by their high agglomeration, low compatibility and poor dispersibility due to the large specific surface area and high surface energy. Zhang et al. [111] prepared a kind of TiO_2_/acrylonitrile–styrene–acrylate (ASA) nanocomposite with different loading contents of TiO_2_ via in situ emulsion polymerization, as shown in Figure 6. To reduce the agglomeration of TiO_2_, different amounts of TiO_2_ nanoparticles were dispersed in the reaction monomer (*N*-butyl acrylate) by ultrasonication. After adding surfactant (SDS) and NaHCO_3_ as well as initiator (ammonium persulfate), emulsion polymerization was carried out under a nitrogen atmosphere. To synthesize TiO_2_/ASA latexes, the pre-emulsion of styrene and acrylonitrile, a certain amount of initiator NaHCO_3_ and chain transfer agent (tert-dodecyl mercaptan) were complementally added and polymerized under the same reaction conditions. The results confirmed that in situ emulsion polymerization can improve the dispersibility of TiO_2_ in the ASA matrix. Moreover, the crystal form of TiO_2_ was not affected by the emulsion polymerization process. The introduction of TiO_2_ nanofillers can result in a significant increase in the impact toughness of TiO_2_/ASA nanocomposites and solar reflectance.

Lee et al. [112] prepared a type of stable nano-TiO_2_/polyurethane emulsion via in situ reversible addition fragmentation chain transfer (RAFT) emulsion polymerization. In this study, the TiO_2_ nanofillers were modified by use of a RAFT agent, 2-((butylsulfanyl)carbonothionyl) sulfanyl prpanoic acid (BCSPA), due to the three coordination modes (monodentate, chelating bidentate, and bridging bidentate) between the –COOH group of BCSPA and TiO_2_ surface. The in situ RAFT emulsion polymerization of 2-hydroxyethyl acrylate (HEA)-capped polyurethane macromonomer was carried out in the presence of TiO_2_-BCSPA as a RAFT agent and azobisisobutyronitrile (AIBN) as a radical initiator. This synthetic method of nano-TiO_2_/polyurethane emulsions can achieve excellent dispersion of the TiO_2_-BCSPA nanoparticles in the polymer matrices, which can maintain stability enough not to precipitate after at least 3 months. Ai et al. [113] reported a novel and simple method to prepare film-forming polyacrylate-core/TiO_2_-shell nanocomposites via in situ emulsion polymerization. No surface modification of TiO_2_ particles or addition of functional comonomer was necessary. Firstly, the TiO_2_ nanoparticles were prepared by use of titanium tetrachloride (TiCl_4_) as a precursor. Then, the in situ emulsion polymerization of acrylate monomers was carried out in the presence of cetyltrimethylammonium bromide (CTAB) as surfactant and TiO_2_ nanoparticles. They demonstrated that the CTAB used herein can provide the latex particles with a positive charge, which was employed as the key acting force to help the adhesion and collection of inorganic phase (negatively charged TiO_2_ particles) and organic phase (positively charged polymer balls). The shape of the particles will not be changed after heat treatment due to the presence of the TiO_2_ coating on the surface of the latex particles. Dan et al. [114] prepared a type of PMMA/TiO_2_ nanocomposite particle by grafting PMMA from the surface of TiO_2_ via in situ emulsion polymerization of MMA. The TiO_2_ particles were modified by the silane coupling agents to ensure the formation of covalent bond binding between PMMA chains with TiO_2_. Several factors including the type of coupling agent, the mass ratio of the MMA monomer to the modified TiO_2_, the emulsifier concentration and the initiator concentration were investigated. The TiO_2_ modified by the silane coupling agent containing the vinyl group can achieve the highest grafting efficiency of PMMA from the surface of TiO_2_ particles. Moreover, the obtained PMMA/TiO_2_ composite particles exhibited a very stable colloidal dispersion in organic solvent. Similarly, they [115] also prepared a type of polystyrene/TiO_2_ composite particle by in situ emulsion polymerization in the presence of MPS-modified TiO_2_ particles. Zeng et al. [116] first prepared the organic nano-TiO_2_ by the sol-gel method using tetrabutyl titanate as a precursor and then the nano-TiO_2_ particles were modified by MPS. The MPS-modified TiO_2_ nanoparticles were used in the emulsion polymerization of acrylates to synthesize nano-TiO_2_/polyacrylate composite emulsions with highly stable dispersion and strawberry-like morphology. The results indicated that the addition of nano-TiO_2_ particles can improve the heat resistance of nano-TiO_2_/polyacrylate composite latex films.

**Figure 7:**
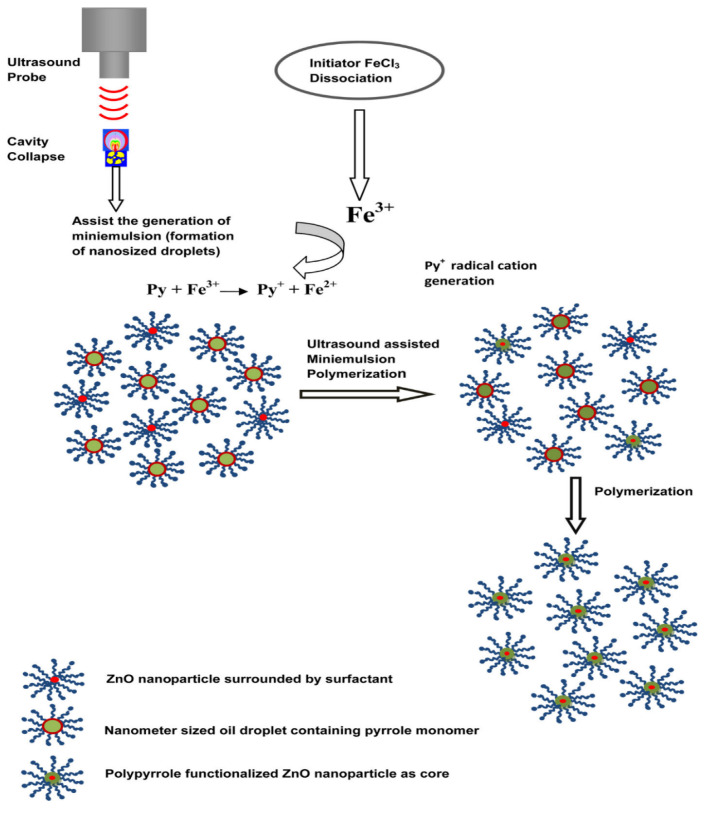
Schematic diagram for the preparation of ZnO/polypyrrole hybrid latex particles via ultrasound-assisted in situ mini-emulsion polymerization, reproduced with permission from American Chemical Society [119].

The multifunctional zinc oxide (ZnO) nanoparticles have drawn considerable attention in recent years [117]. Morsi et al. [118] prepared a type of core–shell nanocomposite of ZnO nanoparticles surrounded by a shell of polyacrylamide via an in situ emulsion polymerization technique. The resulting ZnO nanocomposites were found to possess a broad spectrum of antimicrobial activity. In the work of the Sonawane group, ZnO with high electrochemical stability, good resistivity, and high exciton binding energy and polypyrrole with high conductivity, good environmental stability and easy preparation were chosen as materials to develop polypyrrole-zinc oxide (PPy/ZnO) hybrid nanocomposites for the detection of liquefied petroleum gas. As shown in Figure 7, the PPy/ZnO hybrid nanocomposites were prepared by the use of an ultrasound-assisted in situ mini-emulsion polymerization of pyrrole in the presence of ZnO nanoparticles. During the mini-emulsion polymerization, FeCl_3_ as a dopant molecule dissolved in the water was added to improve the sensitivity to gas molecules. The pyrrole monomer dissolves in oil droplets, which can maintain a small size and stable dispersion due to the synergistic effect of surfactant sodium dodecyl benzene sulfonate (SDBS) and ultrasound. Moreover, the cavitation conditions generated by ultrasound can not only ensure the formation of uniform and nanosized droplets as monomer reservoirs, but also dissociate the FeCl_3_ to initiate the polymerization at the interfaces between nanometer sized oil droplets and the water phase. Moreover, the acoustic cavitation can also produce strong shear and fragmentation of the particles, disrupting the particle aggregation and controlling the size and size distribution of the resulting PPy/ZnO hybrid latex particles with core–shell structure. The uniform nanoscale size of PPy/ZnO hybrid latex particles with stable structure can minimize the response time and improve sensitivity in sensing gas molecules.

**Figure 8:**
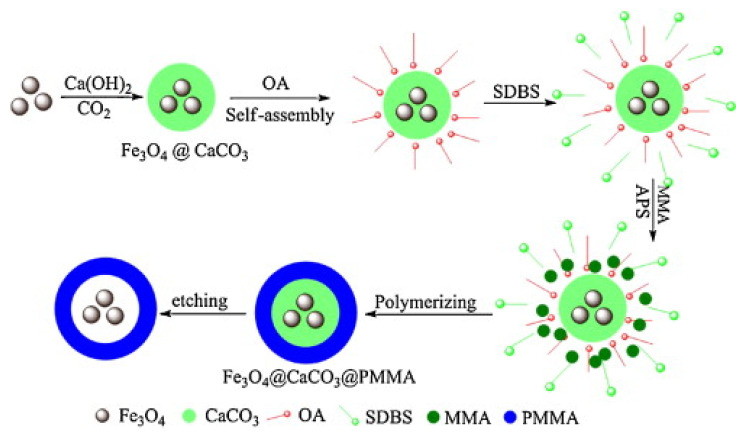
Schematic showing the synthesis of magnetic hollow PMMA latex particles via in situ emulsion polymerization, reproduced with permission from Elsevier [125].

The magnetic nanocomposites have attracted tremendous recent interest and demonstrated a pioneering role in various fields, such as information technology, catalysis, water treatment, biomolecule separation, biosensing, magnetic resonance imaging, magnetic hyperthermia and drug-delivery therapy [120,121,122]. Weller and his coworkers synthesized a type of iron oxide nanocrystal/polystyrene core–shell nanocomposite by in situ emulsion polymerization [123]. The oleic acid-coated iron oxide nanocrystals were treated with PEG-based polysorbate-80 which was employed as the surfactant. The amphiphilic polysorbate-80 can stabilize the outer surface of the nanocrystals, resulting in the formation of a bilayer around the nanocrystals. After swelling with styrene and divinylbenzene in the bilayer around the nanocrystals and starting the radical polymerization by means of a hydrophilic thermal radical initiator, a cross-linked polystyrene shell around the nanocrystals will be formed. Li et al. [124] synthesized a type of CoFe_2_O_4_/polyacrylate nanocomposite by in situ emulsion polymerization of methyl methacrylate (MMA), butyl acrylate (BA) and acrylic acid (AA) monomers. The Triton X-100 surfactant system was employed to stabilize the magnetic CoFe_2_O_4_ suspension and promote the solubility of the acrylate monomer. The CoFe_2_O_4_/polyacrylate nanocomposites were imparted with superparamagnetic behavior. Wang and his coworkers prepared a kind of magnetic hollow PMMA nanocomposite [125]. As shown in Figure 8, they first synthesized core–shell oleic acid-modified Fe_3_O_4_@CaCO_3_ composite nanoparticles. Then, the oleic acid-modified Fe_3_O_4_@CaCO_3_ composite nanoparticles were used in the in situ emulsion polymerization of MMA in the presence of SDBS as surfactant, leading to the formation of Fe_3_O_4_@CaCO_3_@PMMA nanospheres. After etching the template of CaCO_3_, magnetic hollow PMMA nanospheres with strong magnetic properties and hollow nanostructures were obtained, which exhibited great potential for applications in the fields of controlled drug release and targeted drug delivery.

Semiconducting II–VI compounds, typically including materials such as cadmium sulfide (CdS), cadmium selenide (CdSe), cadmium telluride (CdTe), zinc sulfide (ZnS), zinc selenide (ZnSe) and zinc telluride (ZnTe), have received considerable attention due to their applications, such as photoelectric devices, optical limiting, and sensors. The integration of semiconducting compounds into polymers can not only endow specific properties to the polymer/semiconductor composites, but also stabilize the nanocrystals in a solid matrix, leading to a dramatic increase in long-term stability compared with the purely polymer-made devices. However, the performance is dependent on the homogeneous dispersion of inorganic fillers in the polymer matrix. A type of core–shell poly(methylmethacrylate)/cadmium sulfide (PMMA/CdS) hybrid nanoparticle was prepared by surfactant-free in situ and ex situ emulsion copolymerization of MMA with 2-(dimethylamino)ethyl methacrylate (DMAEMA) as auxiliary monomer [126]. This confirmed that the PMMA/CdS nanocomposites prepared via in situ emulsion polymerization can not only offer a more stable place to form the CdS nanocrystals and prevent crystal defects caused by agglomeration, but also obtain monodisperse hybrid nanoparticles and fairly narrow band-gap emission. The core–shell PMMA/CdS nanoparticles synthesized by the post-addition (ex situ) of Cd^2+^ ions showed a wide size distribution and interference fringes in the photoluminescence spectrum due to agglomerates of CdS nanocrystals.

## 3. Hybrid Latex Particles from In Situ Pickering Emulsion Polymerization

**Figure 9:**
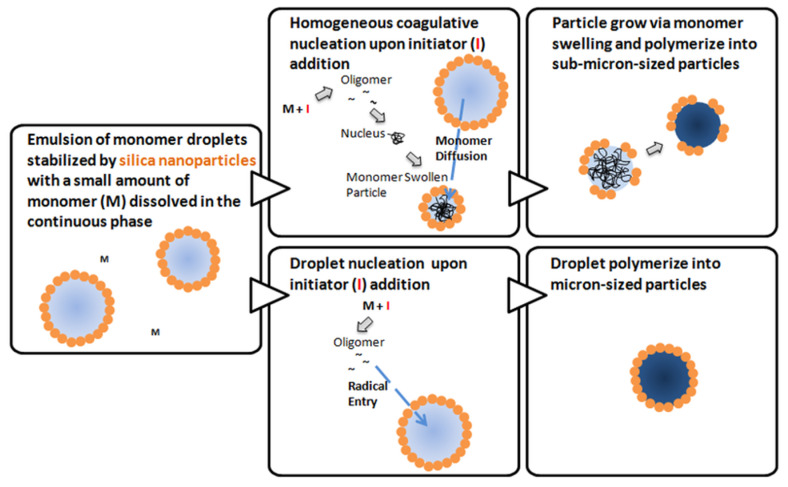
Schematic illustration for possible mechanisms of Pickering emulsion polymerization, reproduced with permission from MDPI [135].

In contrast to the conventional emulsions stabilized by surfactants or amphiphilic polymers, particle-stabilized emulsions known as ‘Pickering emulsions’ have also been reported extensively in recent studies for various applications, due to their superior stability and effective protection of the encapsulated actives within the Pickering emulsion droplets for a long period of time [127,128,129,130,131,132,133,134]. In recent decades, the rapid development of particle synthesis techniques and the discovery of new colloids with tunable surface properties, extended the applications of Pickering emulsions. Unlike surfactant-stabilized emulsions, Pickering emulsions are less likely to cause coalescence, Ostwald ripening, and demulsification owing to the high desorption energy of particles from the interface, endowing emulsions with excellent stability. More importantly, the solid particles serving as emulsion stabilizers cannot only stabilize the emulsion but also impart the emulsion with desired functionalities such as oxidation resistance, UV protection, environmental responsiveness, and even electromagnetic properties. In particular, the in situ Pickering emulsion polymerization has exhibited tremendous potential for synthesizing hybrid latex particles with core–shell morphology. The Pickering emulsion polymerization mechanisms are proposed in Figure 9. The process mainly includes (I) the formation of the Pickering emulsion; (II) the homogeneous coagulative nucleation and droplet nucleation; (III) particle growth via polymerization. After the addition and decomposition of initiators in the aqueous phase, the generated free radicals initiate the polymerization of monomers, leading to the formation of oligomers with radicals. On the one hand, the oligomers will coagulate into nuclei and turn into monomer-swollen particles. The monomer-swollen particles are stabilized by the self-assembled NPs at the interfaces. Subsequently, the particle sizes continue to grow due to the monomer swelling and polymerization within the core. On the other hand, the particles can grow according to droplet nucleation. In this process, the initiated oligomers with radicals will enter monomer droplets and subsequently polymerize into solid cores without significant size growth [135,136]. Homogeneous coagulative nucleation is considered the dominant mechanism, and yields sub-micron-sized hybrid latex particles.

**Figure 10:**
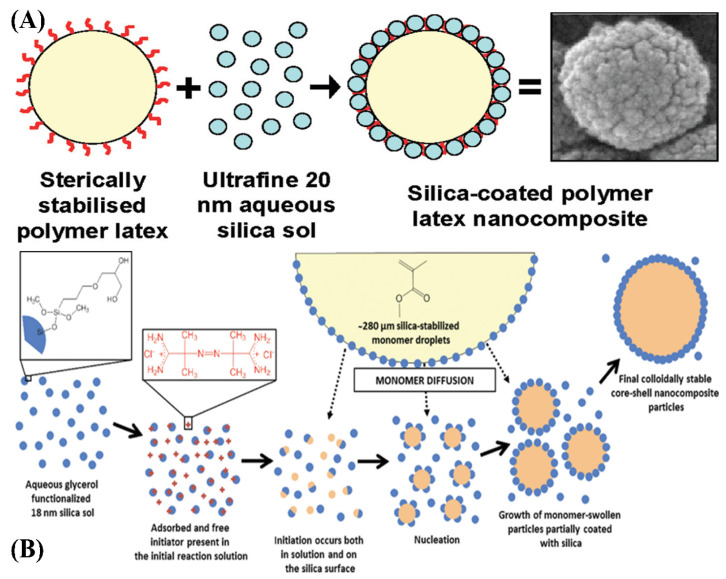
Schematic representation of Pickering emulsion formed by adsorption of ultrafine silica particles onto a sterically stabilized latex (**A**) and preparation process of colloidally stable poly(methyl methacrylate)/silica particles by Pickering emulsion (**B**). Blue circles indicate silica particles and orange regions show polymer, reproduced with permission from American Chemical Society [137,138].

Recently, in situ Pickering emulsion polymerizations have also been widely used to prepare various polymer nanocomposites with hierarchical structures [20,127,130,131,132,133]. In the formation of Pickering emulsions, the colloidal particles are the key component and various particles have been demonstrated to be able to stabilize Pickering emulsions. Especially, the silica nanoparticles with well-controlled size and structure, easy surface modification as well as high resistance to acidic and basic environments have been the most popular materials in the preparation of polymer nanocomposites via Pickering emulsions. The preparation process of polymer nanocomposites via in situ Pickering emulsions is shown in Figure 10A, the small silica particles are adsorbed onto large sterically stabilized polymer latex particles in aqueous solution. Then, the colloidally stable nanocomposites with well-defined raspberry-like “core–shell” morphology can be obtained after in situ Pickering emulsion polymerization of the vinyl monomer [137]. The group of Armes developed and reported a great number of polymer–silica nanocomposites with raspberry-like, currant bun-like, daisy-like, multipod-like, snowman-like or dumbbell-like nanostructures using in situ Pickering emulsion polymerization in the presence of a pre-formed ultrafine silica sol [137,138,139,140,141,142,143,144,145,146,147,148,149,150,151]. For example, they synthesized a series of poly(methyl methacrylate-*co*-n-butyl acrylate)/silica particles by in situ Pickering emulsion polymerization in the presence of an aqueous glycerol-functionalized ultrafine silica sol [138]. As shown in Figure 10B, the cationic AIBA initiator was adsorbed onto the anionic silica particles in the early stages of the polymerization. The adsorbed initiator will decompose to form radicals which cause both surface and solution polymerization of MMA, leading to the formation of hydrophobic patches on the surface of the silica nanoparticles and the resulting nascent nanocomposite particles. After nucleation, MMA diffusing from the metastable silica-stabilized monomer droplets will undergo polymerization inside the growing monomer-swollen nanocomposite particles. On swelling, the silica nanoparticles gradually adsorb onto the bare patches of the nanocomposite surface and fully coat the surface of the nanocomposite particles, leading to full monolayer coverage, high silica aggregation efficiency and good final colloidal stability.

**Figure 11:**
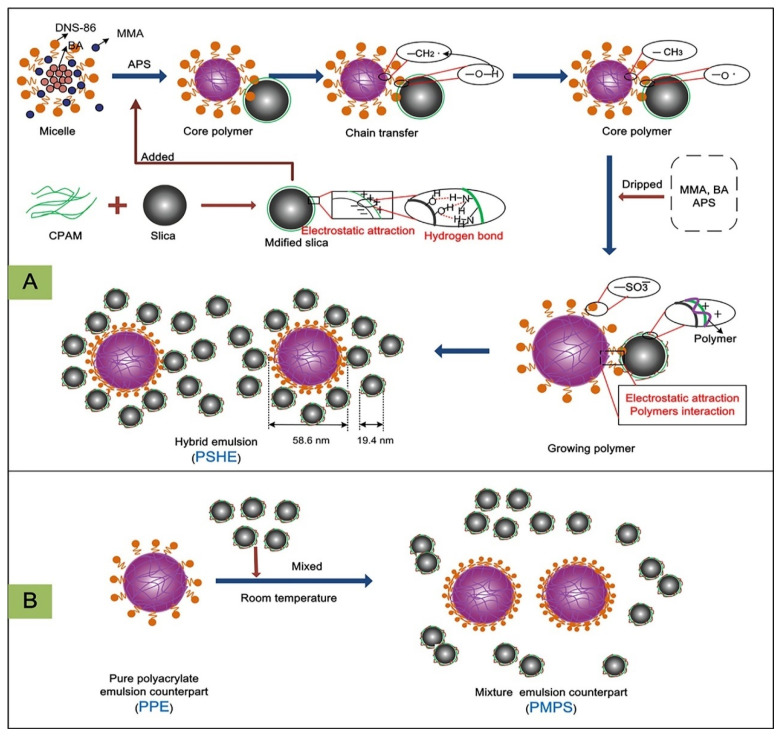
Schematic illustration of the preparation of polyacrylate/modified silica nanoparticles hybrid latex particles (PSHE) via in situ Pickering emulsion polymerization (**A**), and the physical mixture of pure polyacrylate emulsion (PPE) and CPAM-modified silica nanoparticles PMPS (**B**), reproduced with permission from Elsevier [162].

Bao et al. [152,153,154] also prepared polyacrylate/silica nanocomposite latex particles with raspberry-like morphology by in situ emulsion polymerization of acrylate monomers in the presence of AIBA-adsorbed silica nanoparticles. They found that the physical absorption and chemical grafting of polyacrylate onto silica particles took place simultaneously during the in situ emulsion polymerization process. The group of Bon also synthesized a series of clay-armored or silica-armored nanocomposite polymer latexes via an in situ Pickering mini-emulsion polymerization method [155,156,157], in which the laponite clay nanodiscs or silica nanoparticles were used as the Pickering stabilizer. The in situ Pickering emulsion polymerization of MMA was also carried out by the use of nascent silica nanoparticles as Pickering surfactant and cetyltrimethylammonium bromide as cosurfactant in an innovative one-pot route, leading to the formation of the hybrid core–shell organic–inorganic particles with an average diameter of less than 50 nm [158]. Hu et al. [159] applied SiO_2_ nanoparticles as a particulate emulsifier to form Pickering emulsions, which were then added into the melamine formaldehyde prepolymer solution. After in situ polymerization of melamine formaldehyde prepolymer at the interface and outside surface of Pickering emulsion droplets, SiO_2_/poly(melamine formaldehyde) hybrid microcapsules were obtained, which could be employed as an antibacterial essential oil–loaded microcapsule. Wu et al. [160,161] synthesized a kind of waterborne raspberry-like PMMA/SiO_2_ nanocomposite particle via an in situ Pickering mini-emulsion copolymerization of MMA with 1-vinylimidazole in the presence of ultrafine aqueous silica sols as the Pickering stabilizer. They found that the strong acid–base interaction between silica sols and 1-vinylimidazole played a key role in enhancing the formation of long-stable raspberry-like PMMA/SiO_2_ nanocomposite particles. Wang et al. [162] synthesized a polyacrylate/modified silica nanoparticle hybrid emulsion (PSHE) with high silica content via a surfactant-free emulsion polymerization of methyl methacrylate (MMA) and butyl acrylate (BA) in the presence of silica sol nanoparticles, as shown in Figure 11A. The silica sol nanoparticles were modified by cationic polyacrylamide (CPAM) and then used as the Pickering stabilizer. The positively charged silica nanoparticles as the Pickering stabilizer could aggregate around polyacrylate emulsion particles to form a hard shell through electrostatic attraction, hydrogen bonding, and van der Waals forces. As shown in Figure 11B, the pure polyacrylate emulsion (PPE) counterpart was prepared by the same approach without adding modified silica sol. The mixture of latex emulsion (PMPS) was the physical mixture of pure polyacrylate emulsion and CPAM-modified silica nanoparticles. Compared with PMPS, PSHE possessed higher compatibility with polyacrylate and CPAM-modified silica nanoparticles and thus showed better adhesion and dry-wear-resistance as well as increased hardness and impact as well as superior transmittance and superior thermal stability.

**Figure 12:**
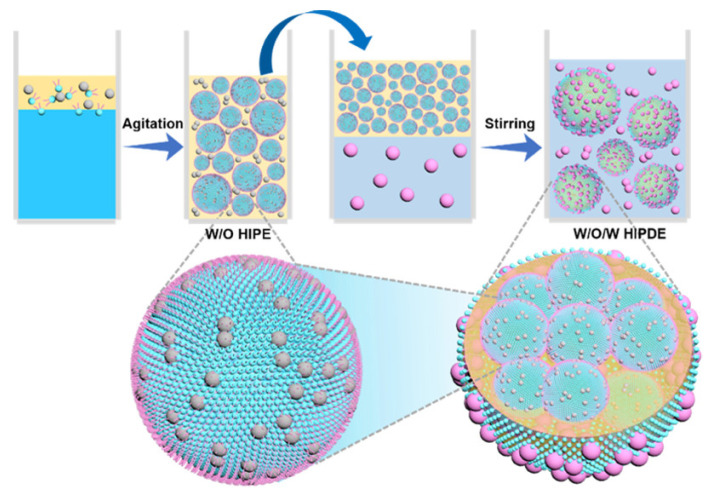
Schematic representation of high internal phase double emulsion latex particles by Pickering emulsion co-stabilized by a biosurfactant lecithin and silica NPs, reproduced with permission from American Chemical Society [163].

Generally, Pickering emulsions are stabilized by various nanoparticles. Nevertheless, it is still challenging to maintain emulsion stability for a long time, because most inorganic NPs without chemical modification are interfacially inert, sometimes leading to desorption from the interface and subsequent demulsification. Thus, an increasing number of studies have focused on the synergistic stabilization of Pickering emulsions by a mixture of surfactants and colloidal particles, combining the advantages of both surfactants and inorganic NPs. For example, Ngai et al. [163] prepared a kind of high internal phase double emulsion of latex particles with tunable structures by Pickering emulsion. As shown in Figure 12, the Pickering emulsion was co-stabilized by simultaneously incorporating biosurfactant lecithin and silica NPs into the emulsion system. The authors systematically investigated the effects of the concentration of lecithin and silica, as well as the pH value of the aqueous phase on the preparation and stability of the emulsions. They demonstrated that lecithin can simultaneously stabilize both W/O and O/W interfaces. The combination of lecithin with silica NPs can be used to stabilize the water-in-oil-in-water (W/O/W) high internal phase double emulsions. Moreover, the hydrophobic interaction or electrostatic interaction between lecithin and silica NPs depended on pH value, which endowed the emulsion with pH-responsive controllable release ability in different environments. Then, the Pickering emulsion templates were used to fabricate functional interconnected porous monoliths and microspheres by in situ Pickering emulsion polymerization of acrylate monomers (e.g., MMA and BA). This study indicated that the cooperation of different surfactants and silica NPs would be a more facile and versatile method to produce high internal phase double emulsion latex particles with a controllable microstructure and desirable properties for a wide range of applications, including homogeneous catalysis, chemical separation, and tissue engineering.

To some extent, graphene oxide (GO) with hydroxyl and carboxylic acid groups on the surface is amphiphilic and thus GO flakes can be located at the oil–water interface. That is to say, GO flakes can be used as a stabilizer in the Pickering emulsion polymerization. A great number of studies have reported the use of GO as a surfactant in in situ Pickering emulsion polymerization [164,165,166,167,168]. For example, Wang et al. [169] prepared a kind of poly(methyl methacrylate) (PMMA)/GO nanocomposite via in situ Pickering emulsion polymerization by use of GO flakes as a stabilizer. The morphology of the prepared PMMA/GO hybrid particles were characterized in detail. The results indicated that the transparent GO sheets were on the surface of the PMMA particles. Moreover, the introduction of GO flakes can inhibit the mobility of the PMMA chains, leading to an obvious increase in the glass transition temperature and activation energies of nanocomposites. Sharif et al. [170] also prepared a kind of PMMA/GO hybrid particle via in situ Pickering emulsion polymerization by the use of GO flakes as stabilizer. They investigated the effect of the size and content of GO sheets on the stability and morphology of colloidal composites. The results indicated that the dispersion of the GO–PMMA particles was unstable, and the coagulation of particles occurred at the early stage of polymerization, when the ratio of GO to MMA was lower than 4 wt%. This may be due to there not being enough nanolayers to fully cover all the emulsion droplets at low GO concentrations.

**Figure 13:**
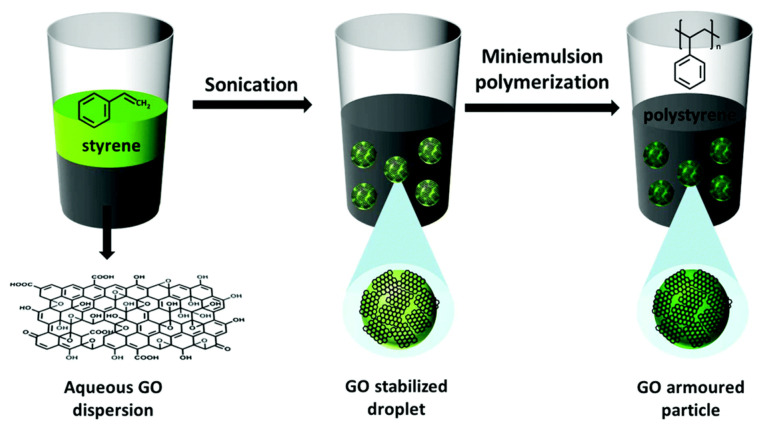
Process and mechanism of Pickering emulsion polymerization of styrene stabilized by GO, reproduced with permission from Royal Society of Chemistry [166].

Zetterlund et al. successfully synthesized polystyrene/GO hybrid particles via in situ Pickering emulsion polymerization of styrene with GO sheets as surfactant in the presence or absence of conventional surfactants [164,165,166]. The nanoscale GO sheets derived from the oxidation and chemical exfoliation of graphite nanofibers, and their morphology and size could be readily tuned by ultrasonication of aqueous GO dispersions. As shown in Figure 13, the stability of styrene/water/GO emulsions can also be optimized on the basis of the investigation of graphene sheet size effects on emulsion stability. They also investigated the influence of monomer type on the stability of Pickering emulsion polymerization using various acrylates and methyl acrylates. They demonstrated that emulsion stability is highly dependent on the nature of the monomer. Monomers such as methyl acrylate and methyl methacrylate with a relatively high polarity can form kinetically stable emulsions in the presence of GO, but there are problems with poor colloidal stability during the subsequent polymerization. However, the monomer with a relatively low polarity such as St can not only generate kinetically stable emulsions in the sole presence of GO, but also undergo and complete the polymerization without significant formation of coagulum/phase separation. In addition, they also found that the Pickering emulsion polymerization of styrene using GO as a sole surfactant suffered from a marked retardative effect on the polymerization rate. Moreover, they demonstrated that the addition of a small amount of the conventional surfactant SDS can dramatically increase the polymerization rate. Fang et al. [168] also prepared polystyrene/GO hybrid particles via Pickering emulsion polymerization using GO as the stabilizer. They found that the pH is an important factor in the stability of Pickering emulsions. A stable Pickering emulsion of GO can be formed at about pH 2 to pH 6. Almost no Pickering emulsion can be formed at pH > 9 or pH < 2. In addition, the effects of different initiators on the morphology of polystyrene/GO hybrid particles in Pickering emulsion polymerization were investigated. The water phase initiator potassium persulfate can produce wrinkled particles while the oil phase initiator AIBN can lead to the formation of spherical particles. Gao et al. [171] investigated the effects of the sonication time, the GO concentration, the oil/water ratio, and the pH value on the stability and morphology of the Pickering emulsions of GO. The longer the time of sonication and higher the concentration of GO used, the more stable the emulsion obtained. The emulsions tended to achieve good stability at intermediate oil/water ratios and low pH values.

**Figure 14:**
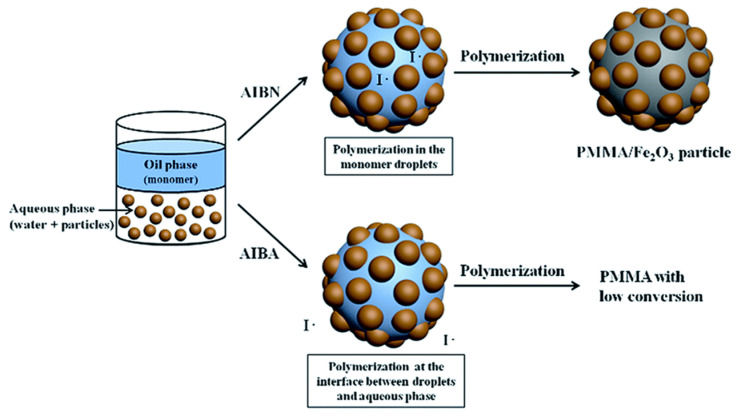
Process and mechanism of Pickering emulsion polymerization stabilized by GO, reproduced with permission from Royal Society of Chemistry [176].

Pickering emulsions can also be stabilized with metal or metal oxide NPs, such as TiO_2_ NPs, ZnO NPs and Fe_2_O_3_ NPs. Wang et al. prepared oleic acid or sodium oleate-modified TiO_2_ NPs, which were used as a Pickering stabilizer in the Pickering emulsion polymerization of styrene. The TiO_2_/polystyrene hybrid latex particles with solid or hollow morphology and a well-defined particle size distribution were obtained by the use of TiO_2_ NPs and oleic acid or sodium oleate-modified TiO_2_ NPs [172,173,174]. They demonstrated that the surface structure of TiO_2_ NPs played a key role in the polymerization routes and thus determined the morphology of the final polymer microspheres. The sodium oleate-modified TiO_2_ NPs facilitated the formation of TiO_2_/polystyrene hybrid latex particles with hollow morphology, while the formation of solid structures was related to insufficient modification of oleic acid-modified TiO_2_ NPs. In contrast, the unmodified TiO_2_ NPs were highly hydrophilic and well dispersed in the aqueous phase, leading to a poor stabilization effect for the initial droplets and the coalescence of oil droplets. Chiu et al. [175] combined Pickering emulsion polymerization and controlled/living free radical polymerization by the use of negatively charged zinc oxide/poly(sodium 4-styrenesulfonate) (ZnO/PSS^−^) nanocomposite particles with an average diameter of 20 nm, as an effective stabilizer in Pickering emulsion polymerization. The ZnO/PSS^−^ NPs covered poly(methyl methacrylate)-*b*-poly(butyl acrylate) (PMMA-*b*-PBA) block copolymer latex was successfully prepared. The hybrid latex particles showed excellent long-term stability against either coalescence or sedimentation over several months, which offered attractive applications for industrially relevant topics. Other researchers have studied the behavior of emulsions stabilized by Fe_2_O_3_ particles for the preparation of novel composites. For example, Choi et al. [176] applied magnetic iron oxide (Fe_2_O_3_) particles (<50 nm) as a solid stabilizer to prepare poly(methyl methacrylate) (PMMA)/Fe_2_O_3_ magnetic hybrid particles via Pickering emulsion polymerization of MMA monomer droplets. As shown in Figure 14, the type of initiator would affect the shape of the final products. In the case of the water-soluble initiator of AIBA, the Fe_2_O_3_ NPs were mixed with polymer and the shape of PMMA was not uniform. In contrast, the application of oil-soluble initiator (AIBN) led to the dominant formation of PMMA spheres covered with magnetic Fe_2_O_3_ NPs. They also prepared a kind of core–shell-structured magnetic polystyrene/Fe_2_O_3_ hybrid particle by Pickering emulsion polymerization of styrene using nanosized Fe_2_O_3_ particles as a solid stabilizer [177]. Qiao et al. [178] prepared a kind lignosulfonate (LS)-modified Fe_2_O_3_ NP and then used the LS-modified Fe_2_O_3_ NPs as a stabilizer for the Pickering emulsion polymerization of styrene. The effect of LS-modified Fe_2_O_3_ NPs on the final morphologies of hybrid latex particles was investigated. Due to their amphiphilic properties and electrostatic interactions, LS can serve as the bridge between Fe_2_O_3_ NPs and polystyrene latex particles, leading to the formation of strawberry-like hybrid latex particles with high superparamagnetic and UV-absorption properties. In sum, the in situ Pickering emulsions have become an important tool for the preparation of hybrid latex particles, due to their easy production, high stability, and lack of toxicity, as well as the possibility of tailoring the size, structure and morphology of latex particles.

## 4. Conclusions

As one of the typical and widely used polymer composite materials, hybrid latex particles combine the unique properties of inorganic nano/micro particles with the inherent properties of polymers, and have been widely used in paints, adhesives, binders, carpet backings, paper coatings, textile treatments, pharmaceutics and so on. The simplicity, high-controllability and environmental friendliness of emulsion polymerization make its use extensive in materials science. In particular, the in situ emulsion polymerization, especially the Pickering emulsion polymerization, has become an important tool for the large-scale preparation of hybrid latex particles with well-defined size, structure and morphology. In this review, the strategies and applications of in situ (Pickering) emulsion polymerization for the preparation of hybrid latex particles were systematically summarized.

Although the application of in situ emulsion polymerization and Pickering emulsion polymerization to prepare hybrid latex particles have achieved tremendous progress, they still face many challenges. For example, the aggregation and precipitation of inorganic NPs are often unavoidable during the process of in situ emulsion polymerization, due to their large specific surface area, high density and poor compatibility with polymer chains. Therefore, to stabilize the hybrid latexes, a large amount of emulsifier could be required, especially when high concentrations of highly hydrophobic NPs were employed. Unfortunately, the high content of surfactant can often affect the formation of polymer particles, cause variations in particle sizes in the final latex, and degrade the properties of the polymer in its final use, such as poor water resistance and low mechanical properties of the polymer. In situ Pickering emulsion polymerization stabilized by only inorganic NPs can achieve higher emulsion stability and reduce the use of a high content of surfactant. However, the addition of various NPs can influence the initiation, particle formation and rate of polymerization processes and sometimes lead to chain termination and/or chain transfer reactions during the emulsion polymerization. With the development of materials technology and synthesis techniques, we believe that the in situ emulsion polymerization, especially the Pickering emulsion polymerization, will become a powerful tool for the fabrication of advanced hybrid micro/nanomaterials for various applications.

## Data Availability

The data presented in this study are available on request from the corresponding author.
